# Management of a large basal cell carcinoma masquerading as psoriasis using Mohs and serial excisions

**DOI:** 10.1177/2050313X241304540

**Published:** 2024-12-06

**Authors:** Rahul Nanda, Thusanth Thuraisingam

**Affiliations:** 1 Division of Dermatology, McGill University, Montreal, QC, Canada; 2 Division of Dermatology, Jewish General Hospital, Montreal, QC, Canada

**Keywords:** Basal cell carcinoma, Mohs, Mohs micrographic surgery, psoriasis, reconstruction

## Abstract

Basal cell carcinoma (BCC) is the most common type of skin cancer. The superficial subtype of BCC may present as a scaly erythematous plaque. This case report discusses a large BCC on the left scalp of an elderly patient who was treated for many years as plaque psoriasis. This report also discusses scouting biopsies as a valuable tool for the dermatologic surgeon to aid in cancer mapping. Finally, we describe the use of serial excisions using Mohs micrographic surgery on the advancing edge as a therapeutic option for large BCCs in high-tension areas.

## Introduction

Superficial basal cell carcinomas (BCC) are a unique clinical subtype of BCC. They present as erythematous plaques with variable scaling, sometimes with rolled borders. We describe a case report of a superficial BCC mimicking plaque psoriasis for years, progressing to a size of over 7 cm. This represented an interesting therapeutic challenge. Herein, we discuss the use of scouting biopsies for tumor mapping as well as using sequential partial excisions with Mohs micrographic surgery (which represents the standard of care for large BCCs) to achieve complete tumor removal.

## Case report

A 60-year-old female was referred for a large plaque on the left scalp. The plaque progressed over 10 years prior to referral, managed initially by the referring dermatologist as psoriasis. The patient’s medical history was relevant only for plaque psoriasis. After treatment failure for several years using topical corticosteroids and Vitamin D analogs, the patient underwent a biopsy 3 months before the presentation of a new nodule that had appeared inside the plaque. The biopsy confirmed the diagnosis of BCC, with both a nodular component and a background of extensive superficial BCC.

**Figure 1.**
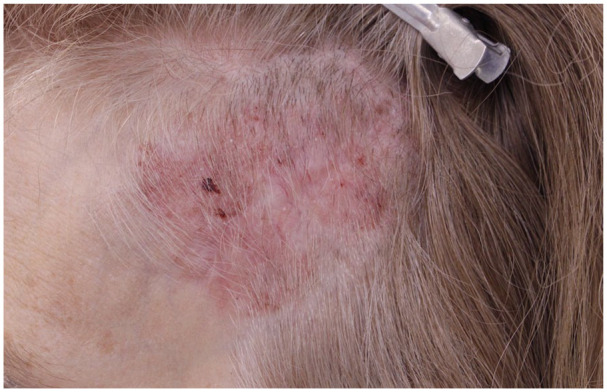
BCC mimicking psoriasis. BCC: basal cell carcinomas.

Clinical examination revealed a 75 × 55 mm^2^ scaly erythematous plaque involving the left temporal scalp extending into the hairline and left temple (Figure 1). At this time, four scouting biopsies were performed at each quadrant of the plaque; all consistent with BCC. We had an extensive discussion with the patient on the treatment options including a single-day Mohs with complete excision and reconstruction with tissue grafting versus serial excision. Given the overall benefit of the smaller surgeries, including cosmetic outcome, reduced pain, and rapidity of healing, serial excision was chosen as the treatment option. After careful planning, multiple serial partial excisions were done of the tumor, starting in March 2021, with verification of the leading margin using Mohs micrographic surgery.

**Figure 2.**
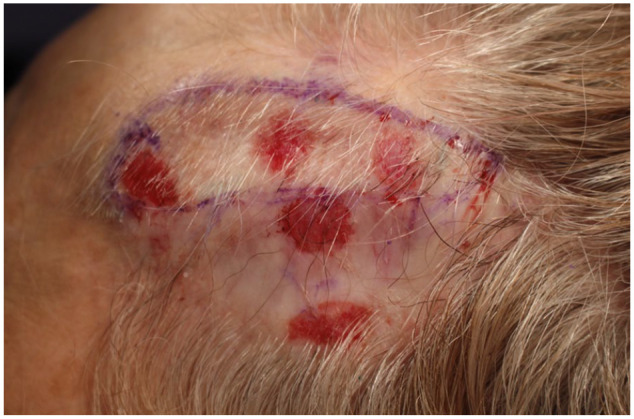
Multiple scouting biopsies done following fourth serial partial excision.

**Figure 3.**
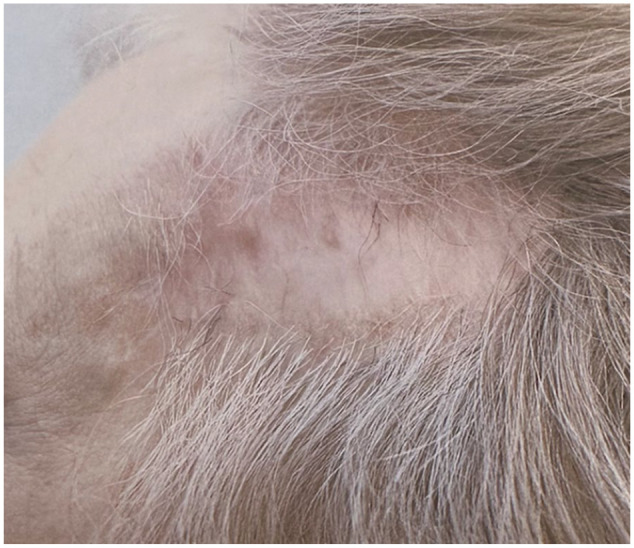
Scarred area following final Mohs treatment and after use of 5-fluorouracil.

The entire visible area of the plaque was completely removed by the fourth partial excision. Of note, each excision was done at approximately 3-month intervals to allow for tissue expansion. Upon follow-up after the fourth excision, there were persistent multifocal areas of well-circumscribed erythema surrounding the scar; these sites were carefully labeled and biopsied (Figure 2). Three of four biopsies showed evidence of BCC, representing either separate primaries or subclinical residual skipped areas. The patient underwent Mohs micrographic surgery once more, encompassing these areas until negative margins were achieved. This final excision was done in January 2023 (Figure 3).

Despite no histological evidence of persistence, the patient was instructed to complete a course of topical 5-fluorouracil adjuvantly and prevent recurrence over her scalp. At 6 month follow-up, no clinical evidence of BCC was noted. She will continue to have yearly follow-up.

## Discussion

Superficial BCC is a distinct clinical and histological subtype of BCC that presents as a well-circumscribed erythematous plaque of variable size, occasionally with a rolled border. Scaling can be present and may be confused clinically with Bowen’s other benign keratotic lesions as well as dermatitis and psoriasis. In fact, multiple case reports demonstrate superficial BCCs masquerading as psoriasis.^1^ Especially on the scalp, the hair should be adequately trimmed before examination. In cases of diagnostic uncertainty or treatment failure, biopsy easily differentiates the two conditions.

Surgery is the treatment of choice for most cases of BCC, with Mohs surgery providing the highest cure rates.^2^ Large tumor size in BCCs is a risk factor for recurrence. Determining the extent of spread can be difficult in clinically equivocal areas of erythema or scaling, especially in patients with extensive sun damage or other papulosquamous/eczematous disorders. Many modalities can be used by the clinician preoperatively to plan an optimal first Mohs layer: dermoscopy, reflectance-confocal microscopy, and scouting biopsies.^3^ The latter can be done preoperatively, or by frozen section on the day of the Mohs procedure.

Scouting biopsies are done in various contexts: recurrent or extensive disease, ill-defined borders, as well as in cases preoperatively where positive results would change guide repair options or require subspecialist involvement (e.g., oculoplastics).^4^ In our case, we performed scouting biopsies to determine the extent of this large skin cancer to better inform the patient of possible repair options. Biopsies were done at each quadrant preoperatively, approximately 1 cm lateral to clinically evident margins, as well as in areas around the primary lesion where there was non-specific erythema or scaling, to exclude skip lesions or other primaries.

The purpose of performing serial partial excisions over months is to take advantage of progressive tissue expansion, which is a process that takes advantage of stress relaxation. The latter, a consequence of tissue creep, is a biomechanical property of skin whereby stretched skin, over time, remodels such that there is less force required to maintain the stretched state. Serial excisions lead to a gradual tissue expansion to close large defects where the wound edges would otherwise not approximate given the high tension of the scalp (barring the use of tissue expanders).^5^ Mohs micrographic surgery to verify both deep margins, as well as margins of the leading edge, were done for the first four excisions, and for the final partial excision, Mohs was done to assess all margins.

Large BCCs require meticulous follow-up to assess for recurrence, especially at scar sites. Various adjuvants can be considered for superficial BCCs, including topical imiquimod, topical 5-fluorouracil, photodynamic therapy, and radiation therapy, among others.^1^
